# MXene−Graphene Oxide Heterostructured Films for Enhanced Metasurface Plasmonic Biosensing in Continuous Glucose Monitoring

**DOI:** 10.1002/advs.202410376

**Published:** 2024-11-21

**Authors:** Rui Li, Hongli Fan, Youqian Chen, Shaoping Yin, Gang L. Liu, Yanan Li, Liping Huang

**Affiliations:** ^1^ College of Life Science and Technology State Key Laboratory for Diagnosis and Treatment of Severe Zoonotic Infectious Diseases Huazhong University of Science and Technology 1037 LuoYu Road Wuhan 430070 P. R. China; ^2^ School of Pharmacy Jiangsu Provincial Engineering Research Center of Traditional Chinese Medicine External Medication Development and Application Nanjing University of Chinese Medicine Nanjing 210023 P. R. China; ^3^ School of Food Science and Pharmaceutical Engineering Nanjing Normal University Nanjing 210023 P. R. China; ^4^ Biosensor R&D Department Liangzhun (Wuhan) Life Technology Co., Ltd. Wuhan 430070 P. R. China

**Keywords:** glucose monitoring, heterostructured material, metasurface plasmonic sensor, microfluidics, MXene

## Abstract

Non‐invasive biosensors have attracted attention for their potential to obtain continuous, real‐time physiological information through measurements of biochemical markers, such as one of the most important—glucose, in biological fluids. Although some optical sensing materials are used in non‐invasive devices for continuous glucose monitoring (CGM), surface or localized plasmon sensing material are seldom applied in CGM owing to modest sensitivity and bulk sensing apparatus. Herein, a metasurface (MGMSPR) biosensor based on the metasurface plasmon resonance chip modified with heterostructured Ti_3_C_2_ MXene−Graphene oxide (MG) is reported, which potentially enables ultra‐sensitive glucose detection. The sensor consists of a dual‐channel microfluidic device integrated with silver mirror enhanced MGMSPR chips. Not only does it promote the entry of glucose oxidase (GOD) into the internal pores and enhance the stable fixation of GOD in the membrane, but also the integration of MG material provides a high specific surface area and unique electronic properties, thereby significantly enhancing the sensitivity of the MGMSPR sensor. The detection limit of MGMSPR biosensor is 106.8 µm. This pioneering approach opens new avenues for monitoring physiological parameters and process analytical technology on an optical platform, providing continuous health monitoring and production process control through optical sensors.

## Graphical abstract

A metasurface (MGMSPR) biosensor based on the metasurface plasmon resonance chip modified with heterostructured Ti_3_C_2_ MXene−Graphene oxide is reported, which enables ultra‐sensitive glucose detection potentially. The sensor consists of a dual‐channel microfluidic device integrated with silver mirror enhanced MGMSPR chips.

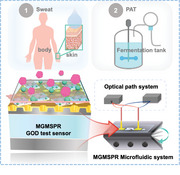

## 1 Introduction

Since the advent of smartphones and other mobile devices, wearable sensors have received a lot of attention because of their ability to provide useful insights about individual performance and health.^[^ ^1^ ^]^ Wearable biosensing technology enables real‐time monitoring of health and disease at the point of care, allowing non‐invasive sampling and analysis of body fluids such as sweat,^[^ ^2^ ^]^ tears,^[^ ^3^ ^]^ and saliva,^[^ ^4^ ^]^ enabling reliable, clinically informative, cost‐effective, and continuous biomedical surveillance.^[^ ^5^ ^]^ Among them, sweat is produced by plasma and interstitial fluid via highly vascularized sweat glands, and it contains a wide range of biomarkers, from electrolytes and metabolites to hormones, neurological markers, and drugs. Human sweat is therefore one of the most challenging frontiers in the field of biomedical sensing,^[^ ^6^ ^]^ and the potential to monitor health‐related biomarkers in sweat has spurred rapid development in the field of wearable sweat sensors. In addition, its easy accessibility and almost uninterrupted sweat volume make it the device of choice for wearable, continuous monitoring.^[^ ^7^ ^]^ Some preliminary studies have shown the value of dynamic changes in various analytes in sweat, including glucose, cortisol, and neuroimmune biomarkers.^[^ ^8^ ^]^ Among the various metabolites, glucose is an important biomarker for the diagnosis and treatment of diabetes, but it fluctuates greatly and depends on the patient's diet and lifestyle. Therefore, sustained glycemic control is important to prevent microvascular complications such as neurologic, retinal, and renal complications that can occur in diabetes. The glucose concentration in the body of diabetic patients is higher than that of normal people, and when diabetic patients sweat, more glucose is excreted with sweat than healthy people, so the glucose concentration in the body of patients can be monitored by analyzing sweat.^[^ ^9^ ^]^ In particular, these devices provide powerful support for personalized medicines by analyzing biochemical markers in sweat, making monitoring activities more convenient and painless.^[^ ^10^ ^]^ On the other hand, continuous glucose monitoring also plays a crucial role in Process Analytical Technology (PAT).^[^ ^11^ ^]^ By enabling real‐time monitoring and control of critical quality attributes, such as glucose concentration, PAT ensures consistency and quality in production processes. In applications such as fermentation and bioreactors, fluctuations in glucose levels as a key metabolite are often a critical indicator of process quality. Therefore, integrating continuous glucose monitoring into PAT systems not only enhances the precision of process control but also minimizes uncertainties in production, ensuring the safety and efficacy of the final product. Therefore, there is an urgent need to develop continuous glucose monitoring systems, providing an effective means for non‐invasive, real‐time health tracking as well as production process monitoring. The application of this technology holds significant potential to improve patients' quality of life and reduce manufacturing costs.

At present, the device for monitoring glucose level in sweat is an innovative technology in the field of health monitoring equipment in recent years, and the research is mainly focused on the development of non‐invasive, real‐time, accurate, and portable sweat glucose monitoring technology. Several types of sensors have been proposed, including biosensing devices based on electrochemistry (EC) and optics.^[^ ^12^ ^]^ The EC sensor uses an external circuit that can generate an electric current to drive the REDOX reaction of the target biomarker for quantitative analysis. However, the design of EC sensors involves at least three rigid metal electrodes and an external circuit, which may have high cost, low stability, and poor anti‐interference ability, preventing their application in clinical Settings. Compared with EC glucose detection methods, optical sensors have the advantages of label‐free, high sensitivity and repeatability, and electromagnetic interference resistance.^[^ ^13^ ^]^ Although Raman and near‐infrared spectroscopy have received a lot of attention as non‐invasive glucose detection techniques, they still need to be further improved to address accuracy and environmental sensitivity issues.^[^ ^14^ ^]^ However, glucose sensors based on surface plasmon resonance (SPR) sensors have the detection advantages of no label, real‐time, simple operation, low cost, and wide measurement range,^[^ ^15^ ^]^ and have greater advantages in convenient detection of glucose.^[^ ^16^ ^]^ However, for target analytes with low molecular weight (<400 Dalton), plasma sensors still face challenges in terms of detection sensitivity.^[^ ^17^ ^]^

Metasurface plasmon resonance (MetaSPR) has been shown to be more portable and have higher sensitivity than traditional angle‐based plasma biosensor designs.^[^ ^18^ ^]^ The MetaSPR chip is a SPR implemented by a subwavelength structure, which is called a grating coupled SPR sensor. Grating coupling is also a simple and convenient method to excite SPR phenomena. As long as the wave vector of a certain order diffused light in the metal‐medium surface direction is equal to the wave vector of the surface plasma wave, the SPR phenomenon will be excited. Silver mirrors have a wide range of applications in optical systems, however, their use in SPR technology to improve sensitivity is still uncommon.^[^ ^19^ ^]^ Silver has a low optical loss and shows a sharper and more intense SPR band in the visible and near‐infrared spectral ranges. These properties make silver an ideal choice for improving the sensitivity and performance of SPR sensors. Inspired by the concept of total reflection of silver mirrors, the chip designed has a specular reflection function, which is similar in function to mirror.^[^ ^20^ ^]^ When light passes through the surface of the MetaSPR chip and reaches the silver mirror, it focuses the beam from the inside to the surface of the chip through strong reflections, ultimately enhancing the coupling of light to the MetaSPR chip. Combined with the Angle independence of MetaSPR chip, the light can be effectively coupled with the surface plasmon in the structure no matter from what Angle of incidence. Silver film exhibits high optical reflectivity and low optical losses, aiding in SPR signal enhancement. Therefore, the combination of silver mirror and MetaSPR technology can realize ultra‐sensitive quantitative detection. Due to the natural properties of optical biosensors, direct labeling free ultra‐sensitive detection of small molecules such as glucose based on MetaSPR biosensors remains challenging, despite its unique nanostructure and silver mirror effect (SME). Therefore, it is also important to improve the surface detection capabilities of MetaSPR sensing technology.^[^ ^21^ ^]^

The improvement of the sensitivity of MetaSPR optical sensing is mainly divided into two main directions.^[^ ^22^ ^]^ One is to use metal nanometers, such as gold nanometers and gold nanorods,^[^ ^23^ ^]^ and the other is to increase the load of the measured object on the sensing surface.^[^ ^24^ ^]^ In previous reports, gold nanoparticles have been studied to enhance SPR signals and improve the detection sensitivity of SPR sensors, but there are limitations for small molecules.^[^ ^25^ ^]^ With the advent of 2D materials, sensors with high sensitivity and low detection limits show great potential in the field of biosensing and medical monitoring.^[^ ^26^ ^]^ At the same time, new nanomaterials are constantly being developed for immobilizing enzymes, which have a dual role in glucose online monitoring.^[^ ^27^ ^]^ Researchers have reported that Ti_3_C_2_ MXene nanosheets with heterogeneous structure and compatibility can be combined with graphene to form 3D structures, thereby promoting enzyme fixation and improving biosensing performance. MXene is an ideal material for enzyme loading and substance detection due to its large surface area, excellent electrical conductivity, and abundant surface groups.^[^ ^28^ ^]^ At the same time, MXene, with its rigid properties, assists flexible graphene oxide (GO) in preparing 3D structures, proving to be a compatible material for the preparation of MXene and graphene 2D materials.^[^ ^29^ ^]^ However, there are few reports of MXene and GO (MG)‐modified enhanced MetaSPR coupling detection of glucose based on online monitoring.

In this study, a metasurface plasmon biosensor based on the 3D material MG enhanced MetaSPR (MGMSPR) was developed for real‐time monitoring of glucose in sweat and PAT. Combined with the SME enhanced MetaSPR chip principle, it reduces scattering losses, provide signal contrast and accuracy, while helping to enhance local electromagnetic fields, further improving MetaSPR signals. MGMSPR biosensor has a lower limit of detection (LOD) and greater specificity than the traditional metasurface. At the same time, the MGMSPR remains label‐free and portable. It is used to detect glucose concentration in sweat samples and can effectively distinguish it from other metabolites. The corresponding biosensor shows high‐performance glucose biosensing ability and realizes glucose detection of complex PAT samples. To our knowledge, this study represents an initial exploration of the dual role of MG hybrid membranes as a highly efficient enzyme and an enhanced optical biosensing platform.

## 2 Results and Discussion

### 2.1 Preparation of MGMSPR Chip and Construction of Microfluidic Platform Device

**Figure 1:**
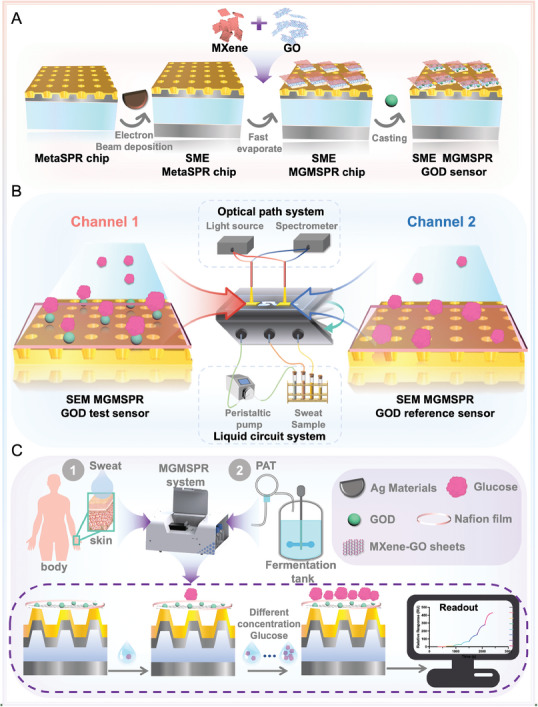
Scheme of the real‐time monitoring MGMSPR glucose quantitative analysis system. A) Principle of the MGMSPR chip to enhance the metasurface sensor by cavity reflection with 2D material. B) Schematic of a reflective dual‐channel sensor for an optical platform. The GOD on the surface of the test chip of the MGMSPR microfluidic platform reacts with the incoming glucose, while no reaction occurs on the reference chip. C) Workflow of an automatic microfluidic device for detecting glucose analytes in sweat and through PAT.

The MGMSPR chip, an innovative label‐free, real‐time, and wafer‐scale biosensor, leverages the unique properties of Metasurface nanoarrays to detect metabolic small molecules. Metasurfaces, which are composed of microscopic periodic structures, possess the capability to manipulate light wave propagation at the nanoscale. As shown in **Figure** **1**A, the MGMSPR chip is designed to enhance the coupling between light and the chip surface by utilizing the strong reflection principle of the SME enhancement. This is achieved by focusing a light beam from within the chip to its surface, thereby creating a MetaSPR surface conducive to the detection of small molecules. The incorporation of 2D materials, such as those with high surface area and specific surface area, significantly improves the sensor's sensitivity. These materials effectively adsorb molecules, thereby increasing the interaction area between the molecules and the sensor surface. The MGMSPR chip integrates 3D MG films, which are fabricated through a mixing‐drying process. The films serve as superior electron transport carriers, further amplifying the local electric field effect generated by the MetaSPR, thus enhancing the sensitivity of chip (Figure 1A). Moreover, by adjusting the composition of MXene and GO sheets, the internal pore size of the 3D hybrid films can be modulated. This adjustable pore size, coupled with the open structure of the MG hybrid membranes, facilitates the penetration of glucose oxidase (GOD) into the internal pores. This design promotes the stable immobilization and retention of GOD within the membrane, which is essential for the development of a reliable sensor. Consequently, the MGMSPR chip, upon incubation with GOD, is transformed into a highly sensitive and stable sensor for the detection of glucose molecules.

To facilitate real‐time monitoring of sweat and PAT glucose levels, the integration of a compact system, known as a Lab‐on‐a‐Chip (LOC), is essential for enabling automated sample injection and real‐time data acquisition. The foundation of our LOC is microfluidics, which pertains to the precise control and manipulation of fluids within networks of submillimeter‐scale microchannels. Therefore, our team has engineered an automated sweat glucose detection system, encompassing a light source, a spectrometer, a pair of MGMSPR chips, an automated flow injection system, fluid conduits, and an optical assembly as depicted in Figure 1B. The dual‐channel MGMSPR sensor is composed of a test chip and a reference chip. The reference channel is added to further provide a reference signal to eliminate the systematic errors in the experiment, such as instrument drift and light source change, so as to improve the accuracy and reliability of the data. The result was an online process for monitoring glucose both in sweat and through PAT (Figure 1C).

As previously discussed, the MetaSPR spectral profile is sensitive to variations in glucose concentration. Consequently, the spectral reflection indicative of glucose concentration is relayed to a computer system for real‐time visualization, with the resultant curve being recorded for further analysis. Following each glucose concentration assay, the sensor chip's surface is meticulously cleaned using deionized water to ensure the integrity of subsequent measurements. The enzymatic reaction catalyzed by GOD transforms glucose into gluconic acid and hydrogen peroxide. The reaction mechanism can be expressed as:^[^ ^16c^ ^]^

(1) $$C_{6}H_{12}O_{6}+O_{2}+H_{2}O\;\overset{\mathrm{GOD}}{\to }C_{6}H_{12}O_{7}+H_{2}O_{2}$$

In this enzymatic cascade, glucose, oxygen, and water are converted into gluconic acid and hydrogen peroxide under the catalytic influence of GOD. GOD's catalytic function is mediated through the reduction of the flavin adenine dinucleotide (FAD) cofactor within the enzyme. The binding of glucose to FAD results in the formation of glucose lactone, which subsequently undergoes spontaneous hydrolysis to form gluconic acid. The FAD cofactor is the active site of GOD, and the loss of this cofactor equates to a loss of GOD activity. During the assay, the binding affinity of the FAD cofactor within GOD diminishes as glucose concentration escalates, leading to a progressive reduction in enzyme activity. This enzymatic activity alteration induces a change in the refractive index (RI) of the solution surrounding the chip, consequently affecting the spectral wavelength. Our optical assessment further correlates the glucose concentrations present in sweat samples with the reflective signals captured by the spectrometer. This approach enables ultra‐sensitive glucose monitoring on the MGMSPR platform, heralding a new era of precision in optical glucose analysis.

### 2.2 The Characteristics and Optimization of SME Sensor Chip

**Figure 2:**
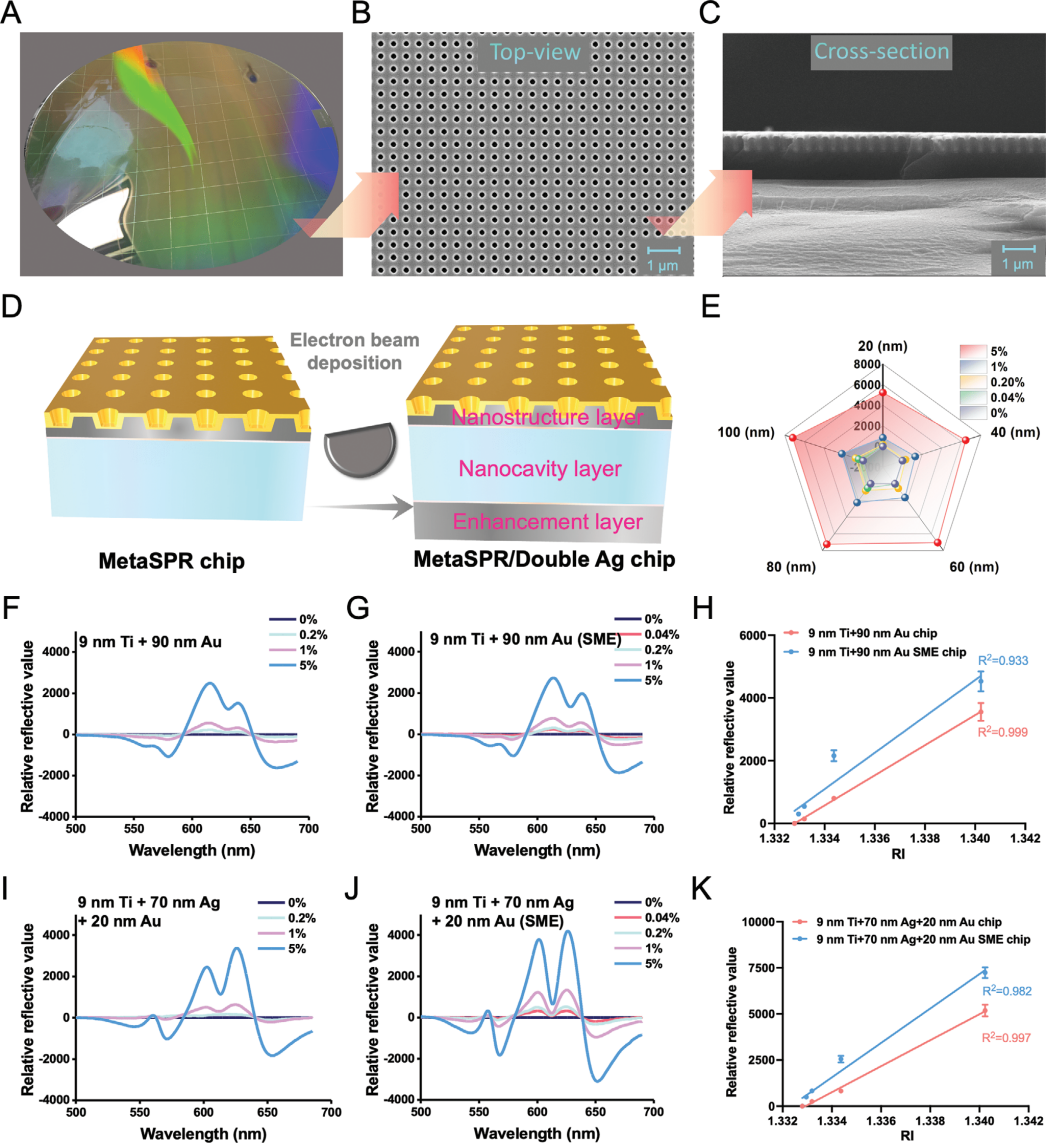
Performance of SME chip sensors. A) Photograph for a metasurface chip. B) Top view SEM image of metasurface chip. C) Cross‐section of metasurface chip SEM image. D) Schematic diagram of preparation of SME chip. E) Relationship between silver deposition thickness and sensitivity. F) Relative reflection spectra of the 9 nm Ti + 90 nm Au chip without SME influence in the 500–700 nm range, with characteristic wavelengths at 605 and 660 nm. G) Relative reflection spectra of the 9 nm Ti + 90 nm Au chip with SME influence in the 500–700 nm range, with characteristic wavelengths at 605 and 660 nm. H) The 4PL regression was fitted to the differential wavelength of sucrose concentration on 9 nm Ti + 90 nm Au chip. I) Relative reflection spectra of 9 nm Ti + 70 nm Ag + 20 nm Au chips without SME influence in the 500–700 nm range, with characteristic wavelengths at 628 and 650 nm. J) Relative reflection spectra of 9 nm Ti + 70 nm Ag + 20 nm Au chips with SME influence in the 500–700 nm range, with characteristic wavelengths at 628 and 650 nm. K) The 4PL regression was fitted to the differential wavelength of sucrose concentration on 9 nm Ti + 70 nm Ag + 20 nm Au chip. The error bars are defined as mean ± SD (*n* = 3).

The MGMSPR optical sensor is characterized by its detection attributes, including its optical response, sensitivity, and repeatability, which are paramount for the molecular monitoring. The design of this platform is tailored to ensure high‐throughput, sensitive, and precise analytical capabilities. **Figure** **2**A is a 12‐inch MetaSPR chip that can be observed to have a visually colored change as a direct result of the collective oscillations of free electrons in the excited metal nanostructure upon light irradiation. This phenomenon responds strongly to the wavelength of the incident light, resulting in a change in the recognizable color as the wavelength of the light changes. The optical chip presented a uniform nanoarray under scanning electron microscopy (SEM, Figure 2B) as well as a cup‐array structure on cross‐sectional SEM images (Figure 2C).

To enhance the sensitivity of MetaSPR chip, the method of steaming metal material and adding silver mirror on the back of the chip was optimized, and the reflection mode was used for detection. Silver (Ag) has excellent optical properties, including high reflectance and good electrical conductivity, which make it ideal as a metal layer in MetaSPR sensors. On the other hand, the silver mirror can act as a reflection layer to enhance the intensity of the electromagnetic field near the chip surface (Figure 2D). When the light wave excites the surface plasma on the metal surface of the MetaSPR chip, the reflection effect of the silver mirror can make the electromagnetic field reflected and enhanced multiple times between multiple metals and silver mirrors, thus enhancing the electromagnetic field interacting with the sample molecules.

First, we evaluated the sensitivity of a chip consisting of adhesive metal titanium (Ti, adhesive metal, and PET film) and SPR classical metal gold (Au), composed of chip (9 nm Ti + 90 nm Au) with a vacuum‐deposited silver film (10 nm Ti + 100 nm Ag + 10 nm Au). Using sucrose as a standard method for testing the sensitivity of MetaSPR chips is both effective and straightforward. Due to its adjustable RI, high chemical stability, and non‐reactivity, sucrose provides a more reliable and stable evaluation system for MetaSPR sensors.^[^ ^30^ ^]^ The performance of bulk RI sensitivity (BRIS) was compared by examining SME and non‐SME chips using sucrose solutions of varying concentrations (0% to 5% w/v) with the RI ranging from 1.3328 to 1.34023, employing a high‐resolution fiber‐optic spectrometer (500–700 nm) across a narrow wavelength range (Figure S1, Supporting Information). In the case of the pure gold chip, the chip without the SME effect could detect a sucrose spectrum at the lowest concentration of 0.2% (w/v), whereas the SME chip exhibited distinct spectral changes at a detection concentration of 0.04% (w/v) sucrose (Figure 2F,G). As the RI value increased, the light intensity at 605 nm increased, while the light intensity at 660 nm decreased. Consequently, the difference in the reflected intensity at two wavelengths (605–660 nm) was utilized to represent the response signal for linear regression analysis. As shown in Figure 2H, the fit between the intensity and RIs for the non‐SME chip (R^2^ = 0.999) and SME chip (R^2^ = 0.933) with 9 nm Ti + 90 nm Au sensor parameters. The LOD of the non‐SME chip and the SME chip were RI = 1.334053 (0.78%) and RI = 1.333143 (0.16%), respectively, with a sensitivity difference of ≈4.9‐fold.

Moreover, the 90 nm Au layer chip was replaced with a 70 nm Ag layer and a 20 nm Au layer, with the Au layer retained to prevent oxidation of the Ag (Figure S2, Supporting Information). As shown in Figure 2I,J, the reflection spectra of non‐SME and SME chips tested with sucrose solutions of different concentrations varied. The specific resonance wavelengths for the metasurface chips were 628 and 650 nm, where sucrose solutions of different concentrations exhibited opposite changes at these wavelengths. Consequently, the reflected intensity at the two wavelength differences (628–650 nm) was used to represent the response signal. Based on linear function, the LOD for the 9 nm Ti+ 70 nm Ag + 20 nm Au parameter non‐SME chip and SME chip were determined to be approximately RI = 1.333776 (0.59%) and RI = 1.332969 (0.047%), respectively, differing by ≈12.6‐fold, with R^2^ values close to 1 (Figure 2K). At the same time, the parameters of 9 nm Ti + 70 nm Ag + 20 nm Au SME chip response values greater than 9 nm Ti + 90 nm Au SME chip parameters. These results indicate that the chip enhanced with a silver mirror possesses superior liquid sensing performance and a greater sensitivity, consistent with the observed increase in sensitivity due to the SME effect in chips with pure gold sputtering parameters. Therefore, the incorporation of a dual silver layer not only improves the sensor's high reflectivity and good electrical conductivity but also enhances the electromagnetic field through multiple reflections and amplifications between multiple metals and silver mirrors.

Subsequently, given that the deposition of different metals can exert varying influences on the SME effect of MetaSPR, a comparison of the thickness of the Ag layer on the reverse side was conducted. A uniform thickness of 10 nm Ti layer was maintained to serve as an adhesive between the substrate and Ag, and a 10 nm Au layer was applied to prevent oxidation of the silver by air. Compared to the Ag layer, Ti and Au layers have a relatively minor impact on the sensitivity of the chip; hence, we chose to optimize the thickness of silver from 20 to 100 nm. The sensitivity of the SME effect chip was evaluated by testing solutions with low sucrose concentrations (0%–5% w/v) (Figure 2E). The performance of metal silver coatings of varying thicknesses was assessed using the reflected intensity at dual wavelength differences. It was found that 80 nm Ag layer was close in performance to 100 nm Ag, indicating that the SME effect reaches a saturation point at a thickness of 80 nm. Consequently, the deposition consisting of 10 nm Ti, 80 nm Ag, and 10 nm Au demonstrated the highest reflected intensity and was capable of clearly distinguishing solutions with low sucrose concentrations. Based on the above studies, the SME chip demonstrated its main optical properties in biomolecular detection, showing promise as a plasma sensor. We chose 9 nm Ti + 70 nm Ag + 20 nm Au SME chip for subsequent research.

### 2.3 3D Material MG Used for MGMSPR Sensor Optical Performance Verification

**Figure 3:**
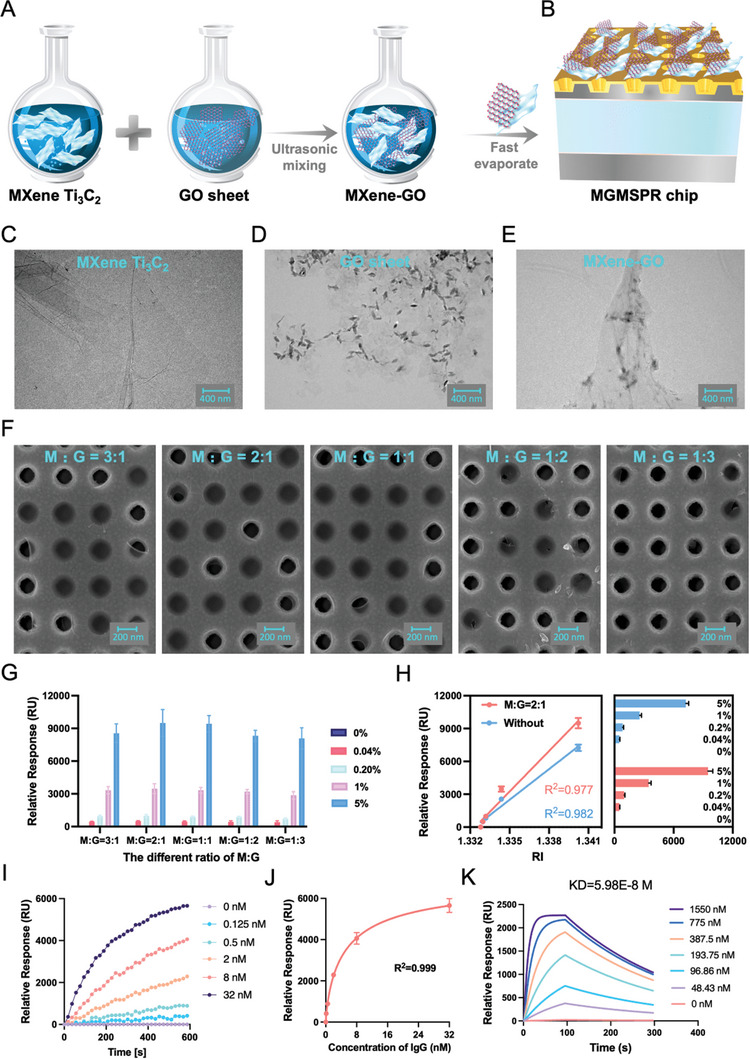
Preparation and performance verification of MGMSPR sensor. A) Preparation of MG films. B) Preparation of MGMSPR sensor. C) TEM of MXene film. D) TEM of GO film. E) TEM of the MG hybrid film. F) SEM of different concentrations of MG hybrid films modified to metasurface. G) Investigation of the correlation between the MG Ratio and MetaSPR sensitivity. H) Analysis of relative response in the 4PL curve for detecting sucrose concentrations. I) Real‐time monitoring of IgG binding curves. J) Wavelength shifts in the 4PL curve for detecting various concentrations of IgG. K) Real‐time monitoring of the association and dissociation interactions between Rapamycin and FKBP12 molecules via MGMSPR sensor. The error bars are defined as mean ± SD (*n* = 3).

Utilizing a straightforward mixing and drying procedure, a 3D MG hybrid membrane was fabricated, as depicted in **Figure** **3**A. The deposition of hydrophilic Ti_3_C_2_ MXene nanosheets onto the Metasurface chip endowed the MG hybrid membrane with an open porous structure and a highly hydrophilic microenvironment, as illustrated in Figure 3B. Examination of the surfaces of MXene nanosheets and GO was conducted using transmission electron microscopy (TEM). The pristine Ti_3_C_2_ film (Figure 3C) exhibited a smooth surface, while the pure GO film (Figure 3D) displayed a wrinkled surface characteristic of GO sheets. In contrast, with the addition of MXene, the stacked nanosheets inside the MG film became more tightly connected to each other (Figure 3E). By simply adjusting the proportions of MG layers, the size of the internal pores was tunable, resulting in varying morphologies on the metasurface chip. As shown in Figure 3F, the 3D membranes crafted from MG film with weight ratios of 3:1, 2:1, 1:1, 1:2, and 1:3 provided a more open architecture. This enhanced the MetaSPR optical response signal and further facilitated the penetration of GOD into the internal pores, as well as the stable immobilization and retention of GOD within the membrane.

To further evaluate the sensing performance of MetaSPR sensors modified with five different types of MG nanosheets as 3D materials, we fabricated MetaSPR sensors for each chip and compared their BRIS characteristics using sucrose solutions with varying concentrations (0%–5% w/v) and a RI range of 1.3328–1.34023. As shown in Figure 3G, all five metasurface chips modified with MG nanosheets exhibited significant spectral changes when detecting a sucrose concentration of 0.04% (w/v). Among them, the chip with MG = 2:1 demonstrated the strongest signal enhancement and the lowest LOD = 1.33285. The MG = 2:1 modified chip exhibited a 36% signal enhancement compared to the unmodified chip (Figure 3H). A linear regression analysis of the MG = 2:1 chip showed a correlation coefficient of R^2^ = 0.977, indicating a strong linear relationship. Therefore, we selected the MG = 2:1 chip for further studies.

To further validate the detection capability of the modified sensor, we selected the 2:1 MG modified chip and performed dynamic analysis and comparison using a molecular quantification platform, aiming to study the real‐time binding curve of Protein A and IgG antibodies. Protein A was immobilized on the sensor chip surface, and different concentrations of IgG (0.125–32 nmM) were introduced into various sensor chips. The real‐time binding curves were obtained by continuously monitoring the corresponding RU over a 10 min period (Figure 3I). As shown in Figure 3J, the 4PL regression analysis demonstrated an ideal relationship between the reflected intensity and IgG concentration (R^2^ = 0.999), with an LOD of 0.055 nmM. Compared to the LOD of 0.37 nmM for IgG detection using unmodified sensors (Figures S3 and S4, Supporting Information), the MG‐modified biosensor achieved a 6.7‐fold improvement in sensitivity. Thus, MG modification significantly enhances the sensitivity for detecting IgG molecules.

Furthermore, the kinetics of small molecules has consistently been a focal point of study within label‐free MetaSPR technology. The molecular interaction between Rapamycin and FKBP12 is characterized by rapid kinetics; however, due to their relatively low molecular weight, such interactions are challenging to detect. To validate the applicability of the MGMSPR system for detecting the kinetic interactions of small molecules, we employed the archetypal model of the Rapamycin‐FKBP12 interaction. The dynamic binding and dissociation phases of the interaction exhibited swift changes, indicative of rapid kinetic behavior, as illustrated in Figure 3K. According to the equilibrium affinity was calculated based on the endpoint of the association phase, the calculated value of dissociation constant (KD) was 5.98E‐8 mM, which aligns with the data obtained using a commercial instrument Biacore (KD = 3.9E‐8 m), as shown in Figure S5 (Supporting Information). Thus, the accurate detection of the fast kinetics of the Rapamycin‐FKBP12 interaction underscores the potential of our developed automated MGMSPR apparatus for widespread use in the study of molecular interactions.

### 2.4 Establishment of Glucose Monitoring Based on MGMSPR Sensor Platform

**Figure 4:**
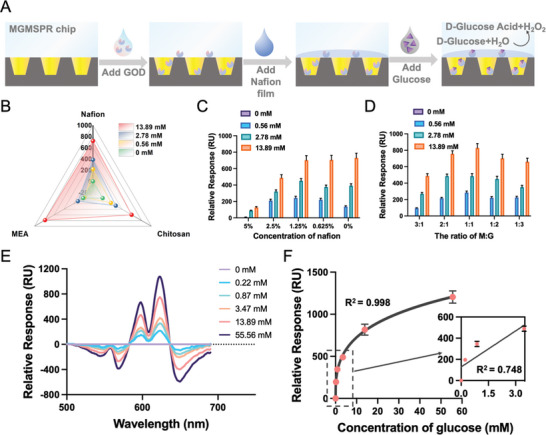
Construction of the MGMSPR glucose chip sensor. A) The principle of MGMSPR sensor for detecting glucose. B) Comparison of glucose detection efficiency using three different methods of GOD modification. C) Comparison of Nafion film modification capabilities at different concentrations. D) Comparison of MG ratio capabilities at different concentrations. E) The differential spectra of glucose detection at various concentrations using the MGMSPR biosensor. F) The relative response of the MGMSPR biosensor as a function of glucose concentration. The error bars are defined as mean ± SD (*n* = 3).

We developed an optical assay for glucose detection utilizing the MGMSPR biosensor. The reaction mechanism of the MGMSPR glucose sensor is illustrated in **Figure** **4**A, detailing the specific steps involved in immobilizing GOD onto the MGMSPR sensor surface and the subsequent glucose detection process. Initially, the MGMSPR chip was thoroughly cleaned with ethanol solution and deionized water, followed by drying. Next, GOD prepared in sodium acetate buffer was applied to the MGMSPR chip surface and allowed to dry, facilitating the immobilization of GOD on the sensing surface. Subsequently, Nafion film was applied to the MGMSPR chip as a protective layer, permitting interaction between glucose and the immobilized GOD. Finally, a spectrometer captured signals corresponding to varying glucose concentrations. In addition, to achieve high‐performance glucose detection equipment, we evaluated three methods for immobilizing GOD: Nafion film‐protected GOD, chitosan film‐protected GOD (Figure S6, Supporting Information), and mercaptoethylamine (MEA)‐carboxylated GOD (Figure S7, Supporting Information). Both Nafion and chitosan are multifunctional polymers with unique chemical and physical properties that make them suitable for immobilizing GOD. Another method involved using MEA to attach GOD to chips, leveraging the chemical reactivity of sulfhydryl and amino groups to form a stable interface.

By testing different concentrations of glucose, the method nafion film immobilized GOD showed the best LOD (0.31 mm, Figure 4B; Figure S8, Supporting Information) and the highest sensor performance for glucose detection compared to the other two modification methods. Therefore, it is considered to be the best candidate modification for the development of MGMSPR‐based glucose sensors for GOD. At the same time, one of the main roles of Nafion as ion exchange membrane, which helps to maintain glucose and GOD charge balance, thus affecting the sensor response time and sensitivity. On the other hand, the amount of Nafion used can have a significant effect on the experimental results. Nafion is used as an ion exchange membrane to help maintain the charge balance of glucose and GOD, thereby affecting the response time and sensitivity of the sensor. Generally, increasing the amount of Nafion used enhances the stability and chemical resistance of the sensor. However, the optical transparency and RI of Nafion are critical factors. Therefore, an appropriate amount of Nafion can ensure good optical transmission performance, while excessive amounts may impair light transmission efficiency. As shown in Figure 4C, the surface of the MGMSPR sensor is modified with five different concentrations of Nafion film, among that Nafion with a concentration of 1.25% has better optical effects and LOD (0.28 mm).

At the same time, 3D MG hybrid films were optimized using a simple mixing‐drying process to develop superior carriers for GOD immobilization. By precisely controlling the weight ratio of MG, the pore size of the MG hybrid membrane can be adjusted accordingly. The results indicate that a 1:1 weight ratio of MG produces a 3D membrane with a more open structure, facilitating the incorporation of GOD into the internal pores (LOD = 0.15 mm, Figure 4D). This enhances the stability and retention capacity of the membrane and improves the optical detection performance for glucose.

Based on the above experiments, the RU for different concentrations of glucose at wavelengths between 500 and 700 nm showed significant optical signal changes as a function of concentration (Figure 4E). RU values at the 628 nm wavelength increased with rising glucose concentrations, indicating a strong correlation between glucose concentrations and response units (Figure S9, Supporting Information). Additionally, the calibration curves obtained for RU values at different glucose concentrations demonstrated a high degree of fit, with R^2^ value of 0.998 (Figure 4F, R^2^ = 0.748 for low concentrations). These results indicate that the response change of the MGMSPR glucose sensor is proportional to the glucose concentration in the sample, validating the feasibility of the MGMSPR biosensor as a glucose monitoring platform.

### 2.5 Real‐Time Monitoring of Glucose in Sweat and PAT

**Figure 5:**
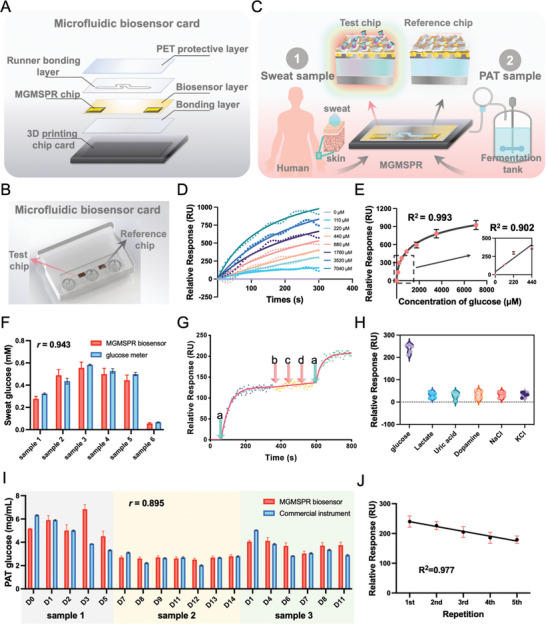
The MGMSPR sensor detects glucose in sweat and PAT. A) Schematic diagram for constructing the MGMSPR sensor ligand and monitoring glucose. B) Photograph of a microfluidic biochip card based on a dual‐channel MGMSPR sensor. C) Schematic diagram for monitoring glucose levels in sweat and PAT. D) Glucose curves in artificial sweat monitored by MGMSPR biosensor at different concentrations. E) The relative response of the MGMSPR biosensor as the 4PL regression for the glucose concentration in artificial sweat. F) Detection of glucose concentration in artificial sweat using the commercialized meter and MGMSPR‐based biosensor. G) Selectivity validation of the MGMSPR glucose sensor (a: 110 µm glucose, b: 100 µmM lactate, c: 10 µMm ascorbic acid, d: 59 µm uric acid). The glucose sensor responds only to changes in glucose concentration. H) Non‐specific validation of the MGMSPR glucose sensor (testing against seven analytes: glucose, lactate, uric acid, Dopamine, NaCl, KCl, sample number = 5). I) Detection of glucose concentrations during PAT using commercially available glucose devices and MGMSPR‐based biosensors. J) Changes in the response of the MGMSPR platform to the number of repetitions are shown. Figure 5E,F,I,J error bars defined as mean ± SD (*n* = 3).

To validate the capability of the MGMSPR sensor in detecting glucose in both sweat and PAT, we conducted real‐time monitoring using artificial sweat as the buffer solution. Microfluidic MGMSPR glucose biosensor chip card from bottom to top by 3D printing chip card, bonding layer, biosensor layer (two MGMSPR chips), runner bonding layer and PET protective layer are integrated (**Figure** **5**A; Figure S10, Supporting Information). As shown in Figure 5B, the microfluidic chip card has two inlets as well as one outlet and two chips for test and reference, and the liquid is pumped to the chip surface by a peristaltic pump for dynamic detection. The Y‐type fiber transmits the light emitted by the light source to the MGMSPR chip, and the reflected signal is received by the spectrometer for visual analysis. The integration of microfluidic with the MGMSPR sensing chip amplifies analytical performance, providing a harmonious improvement in detection power while minimizing the consumption of sample and reagent. As the sample flowed over the surfaces of both chips, the reaction between GOD on the test chip surface and the glucose in the sweat and PAT resulted in RI change. Meanwhile, the reference chip accounted for RI changes caused by other metabolites or ions in the sweat and fermentation broth (Figure 5C).

A series of glucose concentration profiles achieved ultrasensitive detection within 10 min, with clearly distinguishable results within 100 s (Figure 5D). For optimal results, we selected a dynamic test duration of 10 min for the entire biosensor process, allowing users to adjust the reaction time according to their needs. According to the dynamic curve, the average value of the last five data points was taken as the RU value, resulting in a bar graph depicting different concentrations glucose (Figure S11, Supporting Information). The bar graph shows that the response value increases with the sample concentration, consistent with the enzymatic REDOX reaction principle between glucose and GOD. Fitting the standard curve using a 4PL regression also showed a strong correlation between the response signal and different concentrations of glucose, with R^2^ value of 0.993 (Figure 5E, R^2^ value of 0.902 for low concentrations). The LOD is 106.8 µm.

To verify the accuracy of the sweat glucose sensor based on MGMSPR biosensor technology, we detected glucose levels in six standard samples using a commercial blood glucose meter. As shown in Figure 5F, the results indicate that the sweat glucose values obtained by the MGMSPR sensor closely match those measured by commercially available glucose meters. In the comparison between the MGMSPR sensor and the commercial detection equipment, a correlation coefficient of r = 0.943 indicates a strong positive correlation. In addition, changes in sweat glucose levels correlated well with changes in blood glucose levels measured by commercial glucose meters, confirming the reliability and utility of our MGMSPR biosensor for glucose detection.

After monitoring the efficacy of reaction between glucose and GOD, we continue to study the sensor detection selectivity. Sweat contains abundant biological markers, including metabolites (glucose, lactate, alcohol) and a few small molecules (such as cortisol, urea, and tyrosine).^[^ ^1^, ^7^ ^]^ To solve this problem, we add the sweat of other metabolites (100 µMm lactate, 10 µMm ascorbic acid, 59 µMm uric acid) to the buffer for dual channel deduct (Figure 5G). According to the selectivity analysis, a significant change in RU was observed only when 110 µMm glucose was added, with no obvious change upon the addition of other metabolites. Upon adding another 110 µMm glucose, the sensor RU value changed again. This demonstrates that the MGMSPR glucose sensor platform exhibits excellent selectivity for glucose detection. In addition, to test the specificity of the MGMSPR biosensor, we compared the sensor of glucose and other five different solutions, including sweat metabolites (lactate, uric acid, Dopamine, NaCl, and KCl) response, as shown in Figure 5H. The maximum response of the sensor to lactate, uric acid, Dopamine, NaCl, and KCl was only 52 RU, indicating that the glucose sensor has good specificity. This confirms the feasibility of the MGMSPR biosensor in distinguishing glucose in sweat, suggesting its significant potential for further clinical applications.

On the other hand, the MGMSPR sensor presents a promising tool for real‐time monitoring in PAT applications. PAT is a systematic approach to designing, analyzing, and controlling manufacturing processes through timely measurements of critical quality and performance attributes of raw materials, intermediates, and the overall process, ensuring the final product quality. In our study, we selected fermentation broth samples from three different fermentation days for real‐time monitoring as part of the PAT process (Figure 5C). The experimental results, shown in Figure 5I, demonstrate that the MGMSPR sensor exhibited a strong positive correlation (r = 0.895) when compared to commercially available equipment. This suggests that MGMSPR has the potential to serve as a robust and reliable analytical tool in monitoring critical attributes during fermentation processes, contributing to enhanced process control and product quality assurance. The reusability test of the MGMSPR sensor for glucose detection is shown in Figure 5J. It can be observed that the RU value gradually decreases with the increase in repetition/cycle number. Compared to the first measurement, the percentages for the second to fifth measurements are 94.3%, 85.4%, 77.31%, and 74.6%, respectively. This reduction can be attributed to the binding of glucose to GOD, and the dissociation process using PBS is unable to completely release the bound glucose molecules. However, the fitted curve for the number of repetitions exhibits a good linear relationship (R^2^ = 0.977), indicating that the differences caused by the repetition can be accounted for by adjusting the curve.

## 3 Conclusion

This study presents an innovative MGMSPR biosensor based on metasurfaces as an effective platform for monitoring glucose in sweat and PAT. This biosensor enables real‐time, label‐free molecular detection. Using MetaSPR biosensor, a mixture of MXene and GO was modified on its surface, while a silver layer was evaporated on its back, providing multidimensional ultrasensitive detection of analytes. Compared to traditional MetaSPR sensors, the optimized MGMSPR biosensor not only provides a higher specific surface area and increases the local near‐field intensity, but also enhances the MetaSPR signal based on the high reflectivity and low optical loss due to mirror reflection. The experimental results demonstrate that the MGMSPR sensor exhibits a significant increase in sensitivity compared with the traditional metasurface sensor, exhibiting excellent selectivity and stability, which has important application potential in biomedical and human health monitoring.

Furthermore, we developed a system for monitoring glucose in sweat and PAT, consisting of two MGMSPR chips and a microfluidic system. Notably, this method maintained a good curve fitting within the 110–7040 µm concentration range, with a LOD of 106.8 µm. The glucose concentrations in artificial sweat measured by our MGMSPR glucose sensor device closely matched the results obtained using commercial instruments. In conclusion, our research introduces a novel optical detection method based on 3D nanomaterial‐driven metasurfaces for monitoring glucose in sweat and PAT. This approach opens new avenues for the development of wearable sensors aimed at improving patient's daily lives, inspiring future advancements in wearable health monitoring technologies.

## Conflict of Interest

The authors declare no conflict of interest.

## Supporting information



Supporting Information

## Data Availability

The data that support the findings of this study are available in the supplementary material of this article.
