# Supplementary material for: MXene−Graphene Oxide Heterostructured Films for Enhanced Metasurface Plasmonic Biosensing in Continuous Glucose Monitoring
Source: Adv Sci (Weinh). 2024 Nov 21;12(4):2410376. doi: 10.1002/advs.202410376 (PMC11775529; doi:10.1002/advs.202410376)
Supplement: Supplementary file 1 — Supporting Information [file ADVS-12-2410376-s001.docx]

**Supplementary Information**

**MXene−Graphene Oxide Heterostructured Films for Enhanced Metasurface Plasmonic Biosensing in Continuous Glucose Monitoring**

Rui Li, Hongli Fan, Youqian Chen, Shaoping Yin^*^, Gang L. Liu^*^, Yanan Li^*^, Liping Huang^*^

R. Li, H. Fan, Y. Chen, G. Liu, L. Huang

College of Life Science and Technology,

State Key Laboratory for Diagnosis and Treatment of Severe Zoonotic Infectious Diseases,

Huazhong University of Science and Technology,

1037 LuoYu Road,

Wuhan 430070

P.R. china

E-mail: loganliu@hust.edu.cn (G. L.)

S. Yin

School of Pharmacy

Jiangsu Provincial Engineering Research Center of Traditional Chinese Medicine External Medication Development and Application

Nanjing University of Chinese Medicine

Nanjing 210023

P. R. China

State Key Laboratory of Natural Medicines

Department of Pharmaceutics

China Pharmaceutical University

Nanjing 210009

P. R. China

E-mail: ysp0305@126.com (S. Y.)

1. Li, L. Huang

School of Food Science and Pharmaceutical Engineering,

Nanjing Normal University,

Nanjing 210023,

PR China

1. mail: liyanan@njnu.edu.cn (Y. L.), lphuang@aliyun.com (L. H.)

L. Huang

Biosensor R&D Department

Liangzhun (Wuhan) Life Technology Co., Ltd.

Wuhan 430070

P. R. China

**4. Experimental Section**

**4.1 Materials and Reagents**

Ti_3_C_2_T_x_ (MXene) thin layer dispersion and graphene oxide dispersion was obtained from Jiangsu XFNANO Materials Tech. Co., Ltd. (China). GOD type VII (100000 units/g, EC1.1.3.4. from Aspergillus niger), Naﬁon (5 wt %), glucose, sucrose, KCl, NaCl, uric acid, phosphate-buffered saline (PBS) buffer, Tris, and sodium acetate were purchased from Sigma-Aldrich (USA). Lactate was purchased from Shanghai Titan Scientiﬁc Co., Ltd. (China). Protein A and FKBP12 Protein were obtained from Beijing Sino Biological Inc. (China). IgG was purchased from Nanjing GenScript Co., Ltd. (China). Rapamycin was purchased from Shanghai Macklin Biochemical Technology Co., Ltd. (China). Artificial sweat (0.2× PBS, pH 7.4) was purchased from Dongguan Xinheng Technology Co., Ltd. (China). All of the chemicals used for the experiments were of analytical grade and used without further puriﬁcation.

**4.2 Preparation and characterization**

For the metasurface plasmon sensor, nanoblot process was employed to prepare the nanoplasma sensing film. The initial molds were created using laser interference lithography and ion etching to form cone-shaped nanoarrays on silicon oxide sheets. To achieve hydrophobicity, the molds were placed in a vacuum desiccator filled with hexyl silane for 12 hours before replication. Next, NOA-61 was uniformly applied to the mold, and a polyethylene terephthalate (PET) sheet was placed on top. After curing with UV light for 3 min, the PET sheets were carefully peeled off. Subsequently, a thin film layer of 9 nm Ti, 70 nm Ag, and 20 nm Au, as well as 9 nm Ti and 90 nm Au, was deposited on the substrate using electron beam evaporation to fabricate chips with both parameters. Finally, to prepare the silver mirror sensor, an additional layer of 10 nm Ti, Ag layer with different thickness, and 10 nm Au was deposited on the opposite side of the substrate using the electron beam evaporation technique. Using SEM (ZEISS GeminiSEM 300, Germany) characterization of the morphology of all the samples.

**4.3 Preparation of 3D MG Films and the 3D MG Films based MGMSPR biosensor**

Firstly, a mixture of Ti3C2Tx MXene and graphene oxide sheets (1.5 mg/mL) was prepared, in which the weight ratios of Ti_3_C_2_T_x_ MXene and graphene oxide sheets were adjusted to 3:1, 2:1, 1:1, 1:2 and 1:3, respectively. Then, the mixed dispersions were sonicated using an ultrasonic cleaner (40 kHz, 70 W). The MetaSPR chip was washed twice using absolute ethanol and then sonicated in distilled water for 5 min. Next, mix 10 μL the dispersed droplets to the surface of MetaSPR chip and at 60 ℃ in the vacuum drying (20 min). Finally, the obtained chips were called MGMSPR (3:1, 2:1, 1:1, 1:2, and 1:3) sensors, respectively.

**4.4 Sensitivity measurement and affinity determination of MGMSPR sensor**

BRIS analysis was determined by sensor optical properties using solutions with different RI, such as water (RI = 1.3328) and 5% sucrose (RI = 1.3403). Verify the sensitivity, the preparation of 0 ~ 5% sucrose solution and using reflection spectrometer to determine the spectral reflectance of different concentration of sucrose solution. From the spectra of different sucrose solutions, it can be seen that the peak wavelength is redshifted with increasing RI, and the peak wavelength shift has a good linear relationship with the RI of different sucrose solutions. In the differential spectra of different sucrose solutions, the light intensity increases at the peak as the RI value increases.

Subsequently, the MGMSPR sensor is washed twice with isopropanol and deionized water. Following this, the ligand immobilization process begins (e.g., Protein A or rapamycin). The sensor chip is incubated overnight at 4°C with 50 µL of 50 mM 3-mercaptopropionic acid diluted in ultrapure water to introduce carboxyl groups on the surface. The carboxyl groups are then activated by injecting 100 µL of an activation solution containing 200 mM 1-ethyl-3-(3-dimethylaminopropyl) carbodiimide hydrochloride (EDC) and 50 mM N-hydroxysuccinimide (NHS) in 4-morpholineethanesulfonic acid (MES, pH = 6.0) onto the chip. Next, 50 µL of 2 µg/mL protein ligand is added to the activated chip to enable covalent cross-linking via amine coupling. All the above steps are carried out at 25°C, and subsequent experiments are performed under the same conditions. Finally, 100 µL of 1 M ethanolamine (pH = 6.0) and 100 µL of 1% casein (w/v; pH = 7.05) are injected into MES and PBS, respectively, to deactivate unreacted carboxyl groups and block the chip surface. The modified biosensors are stored at 4°C for subsequent analyses.

**4.5 Design of MGMSPR microfluidic chip card and detection platform**

MGMSPR microfluidic chip card consists of two measuring channels (one test channel, one reference channel). In the experimental surface of chip card, it mainly includes two MGMSPR chips (test chip and reference chip) and microfluidic channel. The MGMSPR chip was cut and pasted to the processed dual-channel chip card, and then pasted to the 100 μm double-sided tape flow channel to form the unique sensor chip card. Enclosed microfluidic system and use PET as a protective layer. On the other side of the chip card, there are three flow interfaces, including one liquid inflow hole and two liquid outflow holes. In optical measurements, a platform consisting of a light source, a visible spectrometer, and an integrated fiber probe (Shanghai Ruhai Optoelectronic Technology Co., Ltd.) was used to obtain the reflectance spectra at normal incidence. The platform also equipped with MGMSPR chip card, automatic flow injection device and fluid pipeline system. The liquid pathways in the device, including the PTFE tubing and specific metal coatings on the sensor (such as gold and titanium), have been extensively validated and are known for their excellent biocompatibility, making them ideal for use in medical devices.

**4.6 Prepare MGMSPR glucose sensors and standard determination**

First, the GOD functionalized MGMSPR sensor was immobilized using nafion film. 10 μL of GOD solution (10 mg/mL, dissolved in sodium acetate buffer solution) was added into the center of the chip and incubated in a vacuum oven at 35 ° C for 20 min. The corresponding MGMSPR (3:1, 2:1, 1:1, 1:2, and 1:3) glucose biosensors were prepared by coating 10 μL of 1.25% Nafion solution (dissolved in methanol). Secondly, the MGMSPR sensor was functionalized by immobilized GOD with chitosan film. The above experiment of nafion film replacement for chitosan film, other steps are the same. Finally, the surface of MetaSPR was immersed in MEA solution with concentration of 10 mM for 10 h. After that, the air-dried probe was put into GOD solution for a certain time, and the carboxyl group (-COOH) in the GOD could combine with the free amino group (-NH2) on the probe surface to realize the combination of the GOD and the sensing probe. The biosensor was stored at 4 °C until use. The specific response of the GOD-functionalized MGMSPR/MetaSPR sensor was used to detect different concentrations of glucose standard. The GOD can oxidize glucose into gluconic acid and hydrogen peroxide.

**4.7 Performance evaluation of MGMSPR glucose sensor**

To evaluate the specificity of the MGMSPR-based biosensor, the microfluidic chip was injected with a series of metabolite solutions of 100 μM lactate, 10 μM ascorbic acid, and 59 μM uric acid. After that, real-time monitoring was performed using an optical reflection platform. Artificial sweat was used as diluent and mixed with 110 μM to 7040 μM glucose. To verify the accuracy and reliability of the MGMSPR glucose sensor, a commercial glucose meter was used to measure human sweat concentration. For the analysis of fermentation broth using the MGMSPR platform in PAT, the samples are first centrifuged and then diluted 10-fold before being introduced into the system. It is necessary to initially use the sample from D0 sample to calibrate and correct the background concentration. After this correction, the actual concentrations of subsequent samples can be calculated accurately.


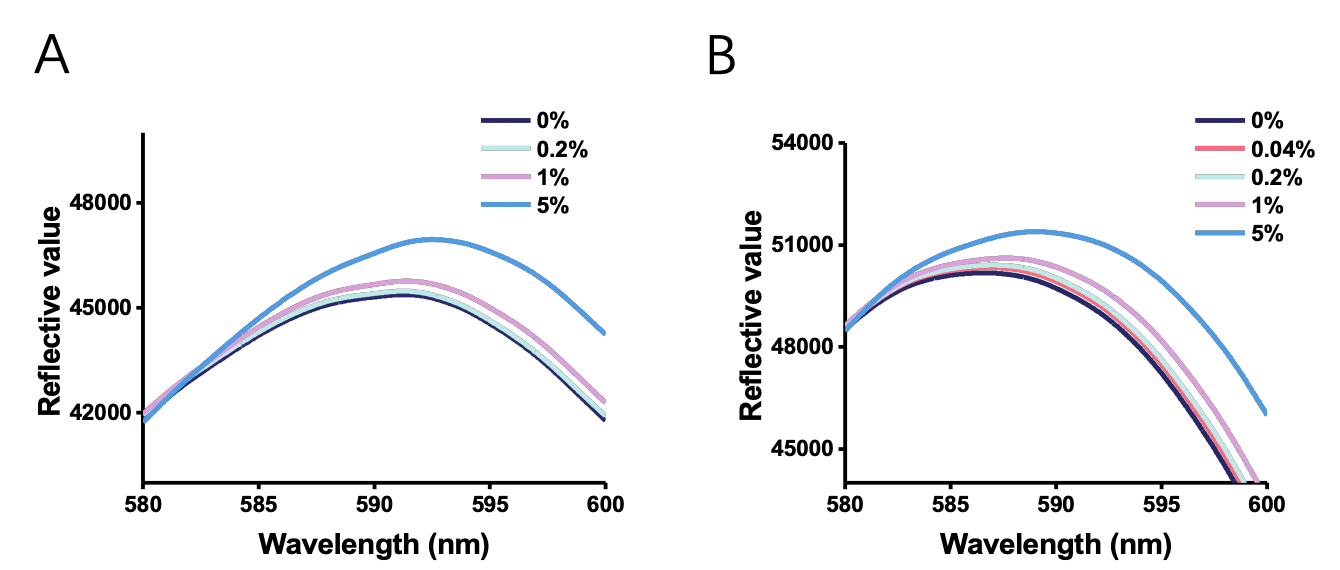
**Figure S1.** A. Original spectra of different concentrations of sucrose detected by chip 9 nm Ti + 90 nm Au (Non-SME effect). B. Original spectra of different concentrations of sucrose detected by chip 9 nm Ti + 90 nm Au (SME effect).


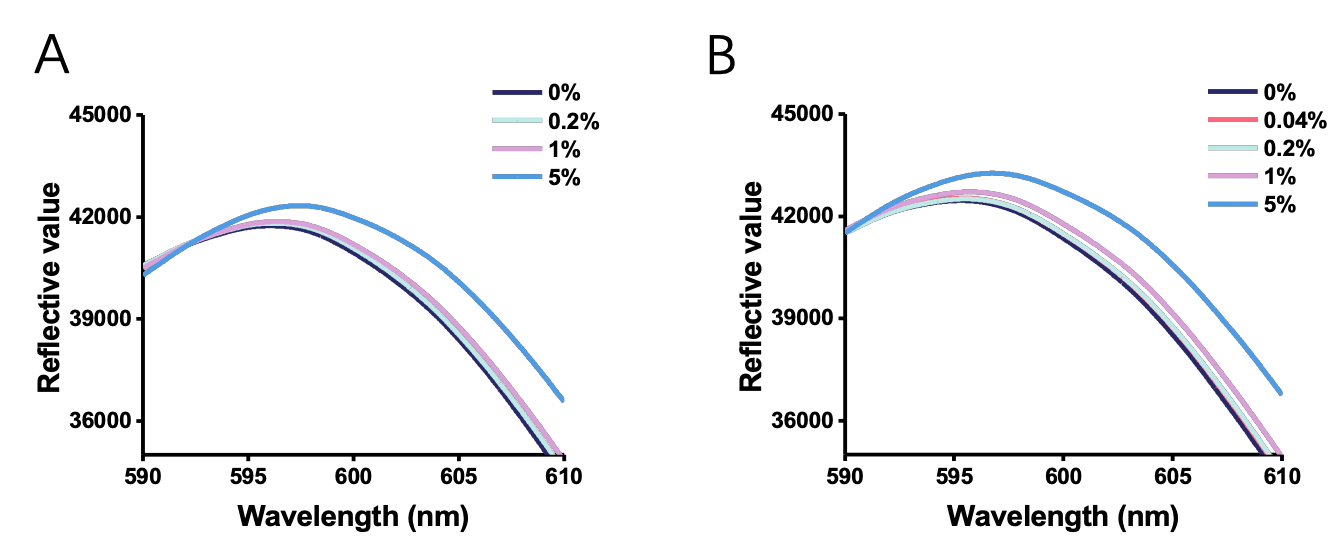


**Figure S2.** A. Original spectra of different concentrations of sucrose detected by chip 15 nm Ti + 90 nm Au (Non-SME effect). B. Original spectra of different concentrations of sucrose detected by chip 9 nm Ti + 90 nm Au (SME effect).

**Figure S3.** Real-time monitoring of IgG binding curves without MG modification. The 4PL fitted curve was used to detect the wavelength shift of different concentrations of IgG.

**Figure S4.** The wavelength shifts of the 4PL curves for different concentrations of IgG were detected by using a chip that was not modified with 3D MG materials.


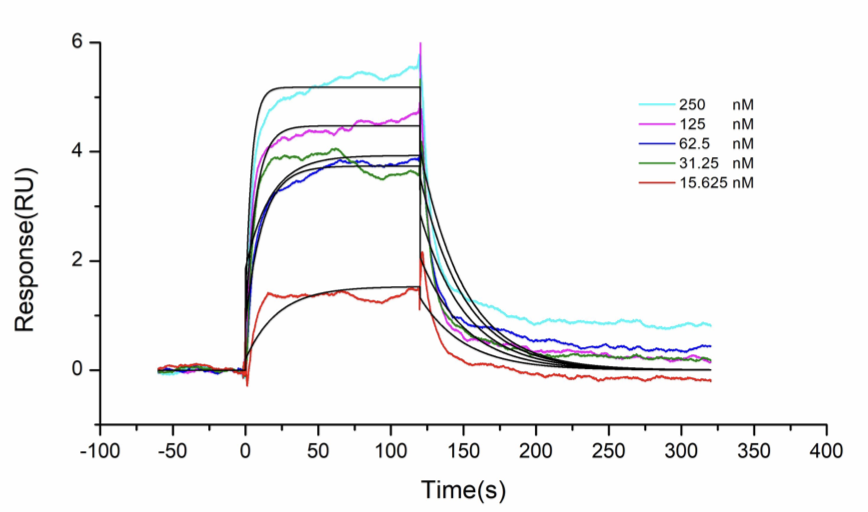


**Figure S5.** Real-time monitoring of the association and dissociation interactions between Rapamycin and FKBP12 molecules using a commercial instrument Biacore.


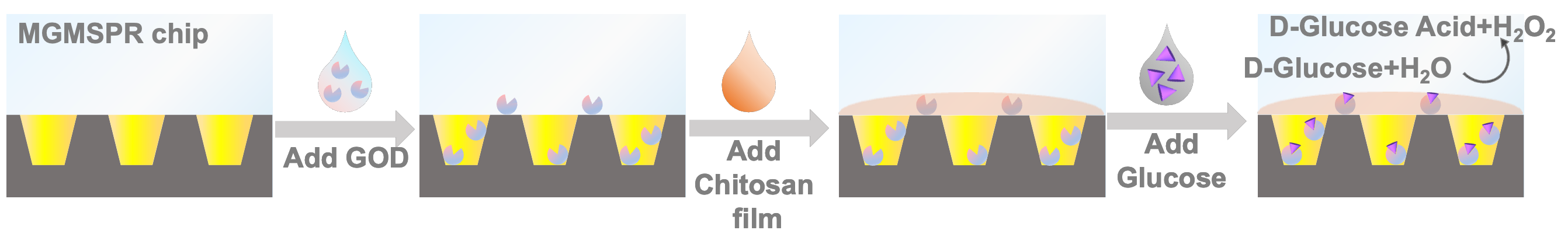


**Figure S6.** Principle of glucose detection based on chitosan fixed GOD MGMSPR sensor.


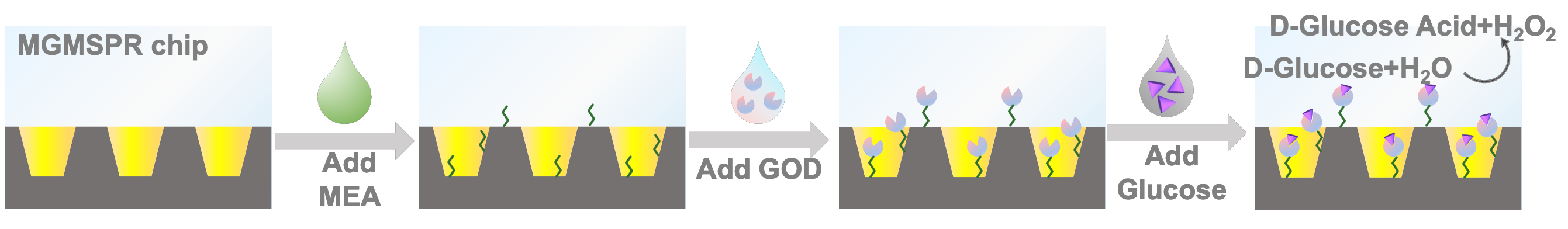


**Figure S7.** Principle of glucose detection based on MEA coupled GOD MGMSPR sensor.


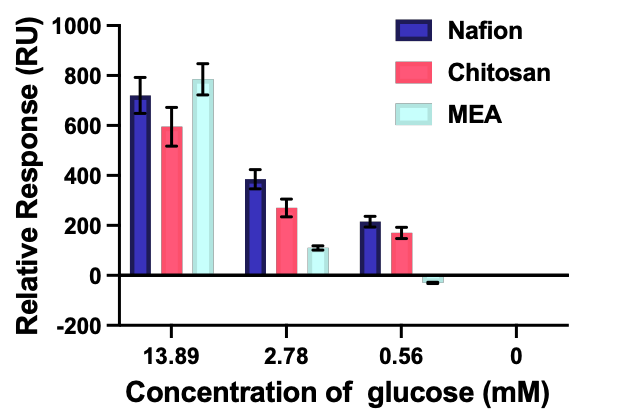


**Figure S8.** Bar graph comparing the glucose detection efficiency of three different GOD modification methods.

**Figure S9.** Bar graph of different concentrations of glucose detected using the MGMSPR biosensor.

**
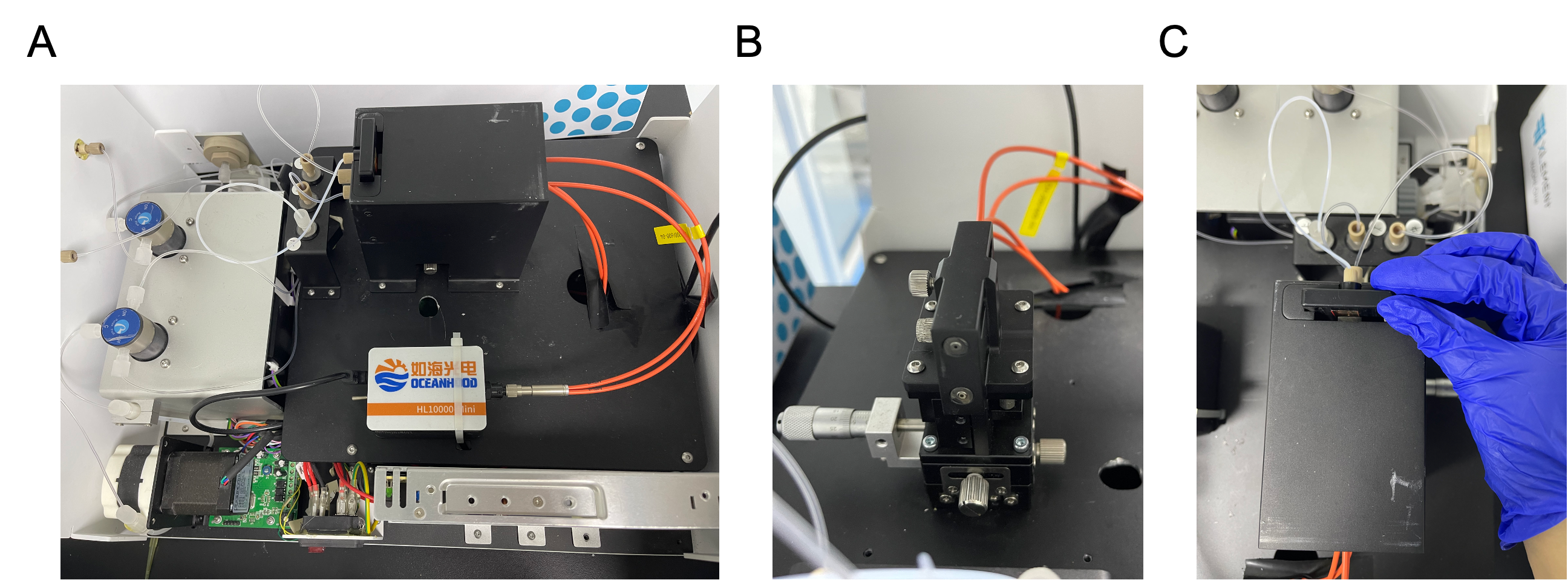
**

**Figure S10.** The MGMSPR sensor is used for real-time glucose monitoring, with: A. Internal components of the device. B. Optical path system. C. Microfluidic cartridge slot.

**Figure S11.** Bar graph of different concentrations of glucose detected using the MGMSPR Microfluidics biosensor.
